# Greener Grass: The Modern History of Epithelial Stem Cell Innovation

**DOI:** 10.3390/life13030688

**Published:** 2023-03-03

**Authors:** Keshia Pitt, Yoshiyuki Mochida, Makoto Senoo

**Affiliations:** 1 Graduate Program in Molecular and Translational Medicine, Boston University Chobanian & Avedisian School of Medicine, 72 East Concord Street, Boston, MA 02118, USA; 2 Department of Molecular and Cell Biology, Boston University Henry M. Goldman School of Dental Medicine, 72 East Concord Street, Boston, MA 02118, USA; 3 Cell Exosome Therapeutics Inc., 2-16-9 Higashi, Shibuya-ku, Tokyo 150-0011, Japan

**Keywords:** stem cells, epithelia, p63, cultured epithelial autografts, holoclones, repsox, howard green, transforming growth factor beta

## Abstract

The field of epithelial stem cell development has been irrevocably shaped by the work of American scientist Howard Green, whose breakthroughs in stem cell culture methods translated to therapeutic practice. In this review, we chronicle the milestones that propelled the field of regenerative medicine of the skin forward over the last fifty years. We detail the early discoveries made by Green and his collaborators, highlight clinical cases that made life-saving use of his findings, and discuss the accomplishments of other scientists who later innovated upon his discoveries.

## 1. Introduction

Epithelial tissues such as the skin and digestive tract line the outer surfaces and inner cavities of organs, and are comprised of multilayered sheets or a monolayer of cells, respectively, that are held together primarily by E-cadherin-mediated adhesion forces [1]. Epithelial tissues are widespread throughout the body and include the epidermis, the reproductive and urinary tracts, the digestive tract, the respiratory tract [2], the mammary gland [3], the prostate gland [4], the salivary gland [5], the cornea [6], and the lung [7]. The thymus is a crucial immune organ for T cell development and is also comprised of thymic epithelia [8].

The epidermis is a classic example of epithelial tissue and acts as a barrier against the external environment. Tight junctions, a network of particle strands mainly comprised of transmembrane proteins, form a paracellular barrier against epidermal dehydration. This function is mediated by adhesion molecules, of which claudin-1 is the most prominent [9]. Corneocytes are flat, anucleated cells organized into a continuous sheet of lamellar lipid layers known as the stratum corneum, which aids in the regulation of water release [10].

Epidermal components exhibit immune competence against pathogens through Langerhans cells and tissue-resident macrophages that function as antigen-presenting cells, as well as through keratinocyte secretion of cytokines and chemokines to initiate inflammatory responses [11]. Within the epidermis reside Merkel cells, mechanoreceptors descended from the epithelial lineage that are required for response to tactile stimuli [12]. Hair follicles, sweat glands, and sebaceous glands are also present, as are neural crest-derived melanocytes. The secreted melanin granules from melanocytes are responsible for skin color and lend photoprotection to keratinocytes against UV radiation [13].

Epithelial homeostasis is maintained through self-renewal of the basal layer of the epidermis; detachment of proliferating cells from the basal layer precedes terminal differentiation and eventual detachment from the outer surface of the skin [14]. Epithelial stem cells are responsible for epithelial homeostasis within stratified epithelial and glandular tissues, and their self-renewal is governed by the transcription factor p63 [4]. The p63 gene encodes two major isoforms: TAp63, whose structure contains an N-terminal transactivation domain [15], and ΔNp63, which lacks this domain and is responsible for the formation of gross epithelium and maintenance of epithelial stem cell proliferative potential [8]. The stem cell niche varies among epithelial structures. In the skin, it is the bulge of hair follicles and the interfollicular epidermis [16]. Corneal epithelial stem cells reside in the basal layer of the limbus [6], whereas the lung contains several populations of epithelial stem cells that maintain distinct regions such as the bronchioles and alveoli [7], suggesting the existence of at least two distinct niches within the lung.

Our current understanding of epithelial stem cell behavior can be traced through functional milestones: advancements in culture techniques, examination of epithelial cells and tissues under homeostatic and tumorigenic conditions, developmental studies to pinpoint cell population origin, and clinical breakthroughs to improve patient care. In this review, we highlight and follow the impact of those milestones on the field.

## 2. Early Epithelial Stem Cell Innovations

The progressive advancement of epithelial stem cell research (Table 1) cannot be explained without Howard Green: a decades-long pioneer and visionary in the stem cell field whose epidermal research revolutionized regenerative medicine. When he published his 1963 paper with George J. Todaro that chronicled the growth of mouse embryo-derived cells in culture, the science of cell line establishment and inoculation technique was yet in development. The very first immortal cell line, HeLa, had been isolated in 1951 from cervical cancer patient Henrietta Lacks [17], and none of the publications cited by Todaro and Green dated prior to 1940. The concept of a “feeder” layer was already established: a living yet mitotically arrested layer of cells used to promote the growth of other cells plated atop them. One factor in the early characterization of HeLa lines was determining which variants required a feeder layer and which did not [18].

Todaro and Green created the original 3T3-Swiss albino cell line (referred to at the time as 3T3) in 1962 from primary cultures of Swiss mouse embryonic fibroblasts. Their paper detailed the lengthy process of the primary cell culture, which first required affirmation of the correlation between growth rate and inoculation size. The optimal parameters for cell plating density were also established: too few cells resulted in zero population growth, while too many caused cell crowding and impeded growth by contact inhibition. By the 15 to 30 generation mark, the growth rate was consistent and remained so when those parameters were followed.

Upon reaching this milestone, Todaro and Green declared those cultures to be established lines with significant replating viability and cultured them past a hundred generations with no signs of replicative senescence. The pair concluded that high inoculation size favors cell line establishment, and that the threshold at which cell growth ceases is higher in cells that were continually cultured at high densities than in those continually cultured at low densities, as was the case for the 3T3 lines [19]. The original 3T3-Swiss albino cell line would later see further notable subclones, including the pivotal 3T3-J2.

In the twelve years between the 1963 paper and further developments with the 3T3 lines, various additional insights in epithelial tissue development were made beyond the epidermis. Improved understanding of the thymus was underway, with attempts made to determine the origin of thymocytes: lymphoid cells present in the thymus that undergo transformation into T cells. At the time, the debate over this origin comprised contradictory data [20]. One side included British developmental biologist John Beard [21] and instructor of anatomy E. T. Bell [22], and suggested that a local transformation of epithelial cells in the thymic rudiment was responsible for thymocyte origin. The other included pathologist William Bloom [23] and proposed a migration from extrinsic mesenchymal cells. In 1967, analysis of cellular migration in chick embryos by Moore and Owen at the University of Oxford concluded that thymic development was dependent on an influx of extrinsic and uncommitted stem cells from the blood. This influx allowed lymphoid differentiation in the epithelial anlage [20].

Rheinwald and Green published two papers in 1975 that established the potential of the 3T3 cell lines in feeder technology. One of their works discussed establishment of a keratinizing cell line that was derived from a mouse teratoma. At the time, the term teratoma was considered imprecise because it made no assertions about structure or behavior; the notion of subdividing the word for better specificity according to malignant behavior remained in question. The general consensus, however, was thus: a teratoma was a real tumor with malformed development, grew rapidly, almost always contained malignant elements comprised of stem cells (called embryonal carcinoma cells), rarely metastasized, was highly transplantable, and usually killed the host by causing emaciation [24]. The modern definition of teratoma has solidified into a benign germ cell tumor comprised of various somatic tissues that are derived from all three embryonic germ layers and are arranged haphazardly [25]. Random growth of hair, teeth, and bone is common [26].

The clone that would become Rheinwald and Green’s keratinizing cell line was derived from a solid tumor grown by Leroy Stevens, created through the graft of a mouse embryo onto the testis of an adult mouse that was given to Rheinwald and Green [27]. The tumor was minced, its cells plated, and small colonies were eventually isolated that retained an epithelial nature. The cells from those colonies survived over 100 generations in culture and became established as the cell line XA1.2 alongside further subclones. Feeder layers of 3T3 cells were used to aid cloning, as normal fibroblasts did not grow well atop them while the teratoma-derived XA1.2 thrived. Staining of the cells with dyes to mark keratin fibers as well as light microscopy revealed that the clones were capable of keratinization, and electron microscopy showed the stratified epithelium in the flattened cell layers of the colonies. Because the original teratoma showed no stratified squamous epithelium, Rheinwald and Green concluded that the keratinizing cells had to come from either embryonal carcinoma stem cells differentiating in culture or a small amount of differentiated cells present in the tumors. The paper stressed that the keratinizing subclone XB was reliant upon 3T3 cells or conditioned medium to differentiate [28].

The second paper published by Rheinwald and Green focused on the keratinization of non-malignant human diploid epidermal cells [29]. Optimization of the relationship between these epidermal cells and the 3T3 feeder fibroblasts, they reasoned, would bypass the extant difficulty in establishing sufficient epidermal cell subculture. Each colony that originated from a single cell in culture formed a stratified squamous epithelium whose basal cells mimicked the appearance of epidermal basal cells. In a phenomenon also observed during the teratoma experiment [28], the 3T3 feeder fibroblasts notably suppressed the growth of living human fibroblasts in culture. Due to this inhibitory effect by the feeder layer, serial cultivation of the primary samples was theorized to eventually produce pure epidermal cells without human fibroblast contamination [29].

One intriguing note from both 1975 papers [28,29] is the inclusion of hydrocortisone in culture medium. Hydrocortisone is the pharmaceutical name for the steroid hormone cortisol and has a myriad of clinical functions, including treatment for patients with adrenal insufficiency [30] and relative adrenal insufficiency (such as in cases of septic shock and cirrhosis [31]), is an immunosuppressant, and is an anti-inflammatory agent. Though primary culture of the human epidermal keratinocytes described by Rheinwald and Green showed no difference in colony appearance in the presence or absence of hydrocortisone, secondary culture and onward demonstrated adequate colony formation but a lack of normal epithelial appearance and decreased lateral expansion in hydrocortisone’s absence. When hydrocortisone was used in tandem with fetal calf serum, the epithelial cells had the authentic stratified appearance [29].

This epithelial cell stimulation by hydrocortisone was not restricted to epidermal keratinocytes. In 1978, Gaffney and Pigott detailed hydrocortisone use in human mammary epithelial cell culture and found that its pairing with fetal bovine serum (another name for fetal calf serum, abbreviated as FBS) increased cell cluster attachment and colony formation by 2.5-fold [32]. FBS and hydrocortisone are essential elements of cFAD, which stands for complete F12, adenine, and Dulbecco’s Modified Eagle Medium (DMEM). cFAD is the growth medium for human epidermal keratinocytes that Green and his collaborators would optimize over the years [33,34], and remains in use today.

## 3. Burns and Breakthroughs

The science of grafting and transplant remains a continual balance between technological advancements and the limitations of the human body. To push those limitations requires innovation, and such was the case with the concept of an autograft: tissue surgically transferred from healthy flesh to a wound on the same individual. The autograft dates back in Western consciousness to surgeon Jaques-Louis Reverdin [35]; while his 1871 paper is usually credited with detailing its invention, documentation of skin grafts to treat mutilations can be found centuries prior in Sanskrit texts from India [36]. Allograft transplant, meaning tissues taken from a donor and given to a recipient, was first described by John Harvey Girdner to cover a burn wound. The 1930s and 1940s saw the establishment of human skin banking, punctuated by the United States Navy tissue bank in 1949—the first program of its kind in the world [37]. Over the next fifty years, the bank pioneered administrative and scientific standards for tissue collection, preservation, and evaluation that included screening methods and donor criteria for cadaver tissue donors, as well as studies on transplantation immunology and development of immunosuppressive therapies [38].

Though monumental, these achievements centered on extant tissues for skin grafts—whether taken from elsewhere on a patient’s own body or from cadavers. Problems arose when burns were suffered to such a degree that there were simply not enough skin grafts available to cover swaths of wounds. The 1975 Rheinwald and Green papers described above [28,29] laid the foundation for a new solution: culturing human epidermal cells in vitro from skin biopsy samples. The growth of cell colonies from founder keratinocytes, achieved with a mitotically arrested layer of 3T3-J2 cells, allowed for their mass culture; 3T3-J2 cells remained the only cells able to support keratinocytes for the next fifty years. Mass culture, in turn, introduced the possibility of more closely matching cultured keratinocytes to the multiple epithelia that would be required for grafts.

In 1979, Green and collaborators Kehinde and Thomas explored this possibility. Green’s cell medium was further optimized through the additions of epidermal growth factor (EGF) and cholera toxin; the former increased culture growth rate and colony-forming efficiency while the latter increased the levels of cellular cyclic AMP (cAMP). The protease dispase was applied to dissociate confluent epithelia from a petri dish in a monolayer, which allowed its placement upon a transplantable collagen surface. This surface was a clear matrix [33] made of extracted rat-tail tendon [39].

In 1984, this technology was applied to life-saving effect. Two children, each with burns covering over 97% percent of their body area, were biopsied for full-thickness skin samples that were minced to create single cell suspensions. From these, cell sheets between two and eight cell layers thick were grown and lifted with dispase. These cultured epithelial autografts were placed onto gauze and applied directly to the wounds. Within two to three weeks, the epithelium achieved uniform thickness. Development of normal epidermal structures such as the horny and granular layers was confirmed. By 21 to 23 weeks, both patients’ wounds were over 85% healed with roughly half of epithelial coverage comprised of cultured epithelial autografts [40].

This breakthrough reached a stumbling block by the 1990s: burn units reported consistently poor clinical outcomes with this technique for massive full-thickness burns. Graziella Pellegrini theorized in 1999 that these poor outcomes were owed to the loss of holoclones—a term and concept first coined by Barrandon and Green [41]. A holoclone is a large, tightly packed, and morphologically immature [42] colony clonally derived from a single stem cell. Holoclones have high levels of p63 expression and maintain their stemness in vitro [43], yet when cultured long-term, their stem cells begin differentiation into transient amplifying cells. These early progeny form colonies known as meroclones, which maintain a degree of p63 expression and proliferative potential that is lesser than that of holoclones [42]. Single cells from the later stages of transient amplifying cell formation produce colonies called paraclones, which have lost p63 expression and show greatly decreased proliferative potential [44]. In short, preservation of holoclones equates to preservation of stem cells that would otherwise be gradually lost throughout meroclone and paraclone transition.

To maintain the integrity of cultured autografts and increase the potential for post-transplant epidermal regeneration, Pellegrini and collaborators used fibrin to cultivate keratinocytes: fibrin being a natural substrate already abundant in the wound healing bed [43]. The established method of mincing skin biopsy samples into single-cell suspension then plating upon an irradiated feeder layer was followed. The cFAD growth medium for co-culture of 3T3-J2 and keratinocytes was also used; the medium consisted of DMEM, Ham’s F12, FBS, insulin, adenine, hydrocortisone, cholera toxin, triiodothyronine, EGF, glutamine, and penicillin-streptomycin [43]. Though this technique of epidermal keratinocyte propagation would see the addition of one final component in the 2010s, Green’s cFAD medium remained largely unchanged and was applied not just for epidermal research but for the stem cells of other epithelial structures, such as the thymus [8].

Pellegrini’s study was comprised of seven patients from infancy to 30 years of age who suffered full thickness burns on 20% to 45% of their bodies. Fibrin gels were prepared from fibrinogen and thrombin solutions [43], thrombin being the enzyme that catalyzes conversion of fibrinogen to fibrin [45], and used as a substrate for the cultured autografts. Because the basal layer where the stem cells resided maintained continuous contact with the supporting fibrin, the stem cells were protected and their clonogenic potential was preserved. In contrast, the cultured autografts detached with dispase and mounted on gauze (as performed by Green and collaborators) lost over 60% of clonogenic potential by the 36 h mark. Furthermore, this technique did not demand fully confluent cultures before epithelial detachment as the Green protocol did; the ability to apply subconfluent cultures granted logistical flexibility between cell culture and surgical demand. All patients showed a stable and fully regenerated epidermis between 2 and 20 months later, and five of the seven patients did not require further grafts or surgeries after the initial application. Pellegrini concluded that preservation of epidermal stem cell-rich holoclones in cultured autografts led to better clinical outcomes for burn patients [43].

Beyond holoclone loss and autograft detachment woes, poor clinical outcomes reported from burn units were attributed to the lack of a dermal component. Several approaches were developed to combat this problem, one being the application of cryopreserved dermal allografts from cadavers onto the burn wound surface. The immunosuppression caused by thermal damage paired with the lesser immunoreactivity of the dermis (as compared to the epidermis) allowed allograft persistence. The epidermis formed from the allograft was stripped off a month after transplant and replaced by cultured epidermal autografts [46].

Another approach was artificial skin, which faced the twofold task of early wound closure followed by long-range function and biodegradable membrane disposal. In 1979, Silver, Yannas, and Salzman crosslinked bovine hide collagen with the glycosaminoglycan (GAG) chondroitin 6-sulfate, which decreased the pro-coagulant and the platelet aggregation activity of the collagen and allowed its potential use as a blood-compatible vascular replacement material in vitro [47]. Yannas and Burke theorized that such collagen-GAG crosslinking could create a membrane for burn wounds on the skin that allowed resistance to collagenase degradation, elasticity, and porousness [48].

In 1981, Yannas, Burke, and collaborators published on the use of artificial skin, comprised of a collagen-chondroitin 6-sulfate fibrillar dermis and a Silastic (silicone elastomer) epidermis, on ten third-degree burn patients aged from 3 to 60. The human type IV collagen of the dermis was derived from cadaver kidneys. The collagen-GAG dermis was applied to support host cell and vessel ingrowth in patients, while the Silastic epidermal portion was temporary at initial treatment and later replaced with autografts. The extensive wounds on all patients were successfully closed by this method with no infections or inflammatory responses [49].

In a 2000 paper, Horch and collaborators acknowledged the progress that fibrin represented and sought to avoid the physical stress that dispase caused to autografts during cellular release from culture surfaces. Because dermal equivalents did not have blood vessels for transport and might block nutrients from reaching the keratinocytes atop them, Horch detailed an “upside-down” method in which subconfluent human keratinocyte monolayers were put in direct contact with fresh wounds on nude mice such that the collagen faced the external environment.

By day 14, the wounds had decreased in size. By day 21, reepithelialization had taken place; well-vascularized tissue was present beneath the epidermis that stained positive for anti-human HLA, as opposed to negative-staining granulation tissue in the mice that were not given keratinocytes. Horch demonstrated the growth of human keratinocytes on type I collagen without a feeder layer, the minimum amount of keratinocytes needed to reconstitute an epidermis in 14 days (100,000 cells/cm^2^), ease and simplification of graft handling procedures, and the preservation of cell adhesion through the lack of enzymatic detachment by the “upside-down” method [50].

The contributions of the scientists described above, as well as of many more from this era, led to optimization of clinical practice in burn treatment and thus to many saved lives. Developments in this field progressed rapidly as new technology created the potential for deepened understanding of the genes, pathways, and processes that governed epithelial stem cells and epithelial tissue regeneration (Table 2).

## 4. Modern Advancements in Epithelial Stem Cell Culture

The biological role of the transcription factor p63 remained in dispute at the turn of the 21st century. Craniofacial defects, limb truncation, and dramatic loss of all stratified epithelia were known consequences of homozygous p63 gene disruption in mouse embryos, which spurred belief that p63 was responsible for maintenance of stem cell populations in epithelia [5]. A different perspective posited that p63 was a molecular switch, wherein the ΔNp63 isoform functioned as a dominant negative against the TAp63 isoform to prevent keratinocytes from Ca^2+^-mediated terminal differentiation in vitro [51]. This perspective was not consistent with the lack of epidermal phenotype in TAp63-specific knockout mice [52].

Our lab’s 2007 paper examined the effect of p63 on epithelial stem cells in the thymus, a tissue whose epithelial component had been acknowledged for decades [20]; both cortical and medullary thymic epithelial cells were found to share a single bipotent epithelial progenitor [53,54]. To determine whether epithelial stem cells still executed commitment and differentiation programs in p63′s absence, both the development of the whole thymus and the expression patterns of thymic epithelial cells were assessed at earlier time points in wildtype and p63 knockout mouse embryos. At E11, prior to the expression of p63, the thymic anlage of wildtype and p63 knockout showed similar levels of pan-CK, an indicator of primordial commitment into epithelial lineage. At E15.5, the expression level of epithelial lineage markers such as K5 and K8 in thymic epithelial cells was identical between the genotypes; these data indicated that commitment and differentiation of epithelial stem cells indeed occurred in p63 knockout mice [8].

To examine thymic development, we compared p63 knockout mouse embryos to wildtype mouse embryos at E19.5. We found that the knockout presented with thymic hypoplasia [8], which mirrored the defects observed in other epithelial tissues [5]. DNA microarray data showed that in the later stages of embryonic development, the knockout thymus showed more terminally differentiated epithelial cells than immature cells.

We then sought to determine if p63 was required for the proliferative potential of thymic epithelial stem/progenitor cells. To enrich immature thymic epithelial cells, we used rats as a model and we collected holoclones with the Green method. We transduced these rat thymic epithelial cells with a lentivirus that encoded shRNA against p63 transcripts. The shRNA treatment resulted in reduction of p63 protein levels to less than 5% of control cells as confirmed by Western blot. Though the p63-silenced cells and the control cells showed similar percentages of Ki67-positive cells in the early stages of 3D culture (thymospheres), the former developed smaller colonies than the latter and showed a smaller percentage of Ki67-positive cells at later stages [8].

As well as the effect of p63 on thymic epithelial stem/progenitor cells, our paper tested the role of p63 in epidermal stem cells. p63-positive rat epidermal cells generated holoclones, meroclones, and paraclones when grown in clonogenic culture that were similar to the patterns of human epidermal cells in vitro, which enabled their use as a model. Transduction with the lentivirus that expressed shRNA against p63 transcripts showed a 90% reduction of p63 protein levels in these epidermal cells by Western blot, compared to cells transduced with a control shRNA lentivirus. By days 10 and 14, the stem cells with an shRNA-induced reduction of p63 formed far smaller colonies than control cells. Our lab concluded that while p63 is not required for stem cell commitment and differentiation during tissue morphogenesis, it is indispensable for maintenance of the proliferative potential of epithelial stem cells [8]. This knowledge enabled greater understanding of the mechanisms underlying epidermal stratification [55].

To discuss the final component of Green’s epidermal keratinocyte propagation technique, we must first mention two new individuals: Kazutoshi Takahashi and Shinya Yamanaka. Through an exhaustive process of elimination, it was determined that the transcription factors Oct3/4, Sox2, c-Myc, and Klf4 in tandem with each other are able to reprogram differentiated cells into induced pluripotent stem cells (iPS cells) that exhibit the properties of embryonic stem cells. Though a fifth gene called *Nanog* was implicated in this process, it was ultimately found disposable for iPS cell induction and maintenance [56].

As with Green’s cultured epithelial autografts, Takahashi and Yamanaka’s groundbreaking discovery came with key caveats. The viral vectors used for transfer and induction of what became known as the Yamanaka factors were found to increase tumorigenicity and cause aberrant transcription, which hampered potential clinical use [57,58]. Justin Ichida and collaborators posited in 2009 that if small molecules could catalyze the reprogramming of differentiated cells into iPS cells through either direct activation of the Yamanaka factors or compensation for their activity, viral vectors would no longer be needed to do so [59]. Of the four factors, Oct3/4 and Sox2 were especially poignant, not only for being essential to the maintenance of embryonic stem cell pluripotency but for regulating other genes necessary for development [60].

Ichida’s paper focused on Sox2, culturing *Oct4::GFP* transgenic mouse embryonic fibroblasts that were transduced with *Oct4*, *c-Myc*, and *Klf4*. Further, 1 compound from a library of 800 with known pharmacological targets was added to each well of a 96-well plate. Three wells yielded GFP^+^ colonies, which indicated the growth of mouse embryonic stem cell-like colonies in the absence of Sox2. Because the 800-compound screen included the histone deacetylase inhibitor (HDAC) valproic acid in the cell culture medium, further tests were required to see if the small molecule candidates required an HDAC inhibitor to reprogram differentiated cells. From the three candidates, only the transforming growth factor-β (TGF-β) receptor 1 kinase inhibitor E-616452 was shown to form GFP^+^ colonies in the HDAC inhibitor’s absence. For its ability to replace both Sox2 and c-Myc (whose absence reduced tumor formation risk), E-616452 was chosen for further characterization and given the name RepSox—Replacement of Sox2 [59].

TGF-β is a superfamily of cytokines whose receptors are ubiquitous on every cell surface [61]. It has three isoforms encoded by the TGFB1, TGFB2, and TGFB3 genes, and its proteins serve a vast array of functions that include regulation of epithelial cell growth and differentiation [62]. Ichida found that RepSox inhibited the receptor kinase for TGFB1, interrupting the TGF-β signaling pathway in cultures whose cells were only partially reprogrammed by Oct4, c-Myc, and Klf4. Due to this inhibition, Nanog—the fifth gene considered by Takahashi and Yamanaka—was upregulated and drove the completion of iPS cell reprogramming. Ichida theorized that Nanog may repress differentiation signals, and that it might collaborate with Klf4 in a similar fashion to Sox2 to spur the reprogramming of differentiated cells into iPS cells [59].

The connection between RepSox and the work of Rheinwald and Green was made years in the wake of this discovery as our lab investigated the potential of TGF-β inhibition for epithelial cell culture. Before this, the only recourse to stall rapid differentiation of primary keratinocytes was with low Ca^2+^ medium, and even then senescence was evident after only ten days [63]. The efficiency of RepSox for TGF-β inhibition was shown in our 2015 paper on the Wnt/β-catenin antagonist Dact1, which is highly expressed in 3T3-J2 feeder cells and uniquely allows them to support epithelial stem cells. Suppression of Dact1 inhibited the function of 3T3-J2 cells, preventing their ability to support keratinocyte stem cell growth by promoting the overproduction of TGF-β. The suppression of TGF-β by RepSox in non-productive feeder cells with undetectable levels of Dact1 allowed the generation of completely humanized skin grafts, as human feeder cells were enabled to support human keratinocytes for the first time [64].

**Figure 1:**
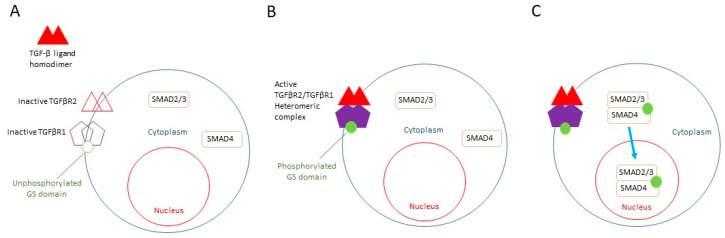
TGF-β signaling pathway. (**A**) Inactive TGFβR2 (open triangles) and TGFβR1 (open pentagons) present on the cytoplasmic membrane with unphosphorylated GS domain (open circle). Unphosphorylated SMAD2/3 and SMAD 4 (rectangular) in the cytoplasm. Unbound TGFβ ligand homodimer outside of the cell (closed triangles). (**B**) Binding of TGF-β ligand homodimer to TGFβR2, resulting in the formation of active heteromeric complex of TGFβR2 and TGFβR1 (closed triangles and closed pentagons). This leads to phosphorylation of TGFβR1 in its GS domain (closed circle). (**C**) Subsequent phosphorylation of SMAD 2/3 complex. Translocation of SMAD 2/3/4 complex into the nucleus.

The knowledge of the TGF-β ligand pathway that leads to the nuclear translocation of SMAD2/3 (Figure 1), the observation of the large fraction of SMAD2/3 in the nuclei of low-proliferative mouse primary epithelial keratinocytes, and the known role of RepSox as a TGF-β inhibitor led to the hypothesis that RepSox could support epithelial keratinocyte proliferation through the suppression of SMAD2/3 nuclear translocation, preventing terminal differentiation [65].

**Figure 2:**
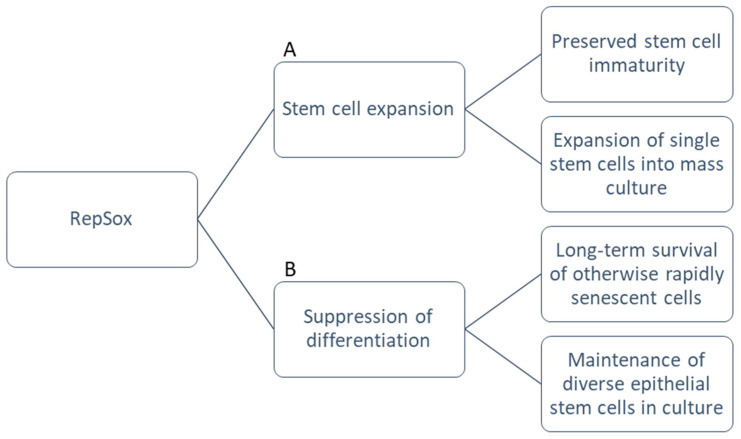
Functional advantages of RepSox. (**A**) RepSox maintains immaturity of stem cell populations and supports clonogenic potential. This allows rapid expansion of single stem cells into mass cultures for large-scale experiments. (**B**) RepSox enables long-term survival of cells without senescence or differentiation in diverse epithelial cell types.

Our data showed that not only did RepSox stimulate proliferative capacity, it enriched p63-positive epithelial stem/progenitor cells derived from the mouse epidermis in tandem with 3T3-J2 co-culture. This allowed for long-term survival of these otherwise rapidly senescent cells. TGF-β signaling thus correlated inversely with the clonal potential of epithelial stem/progenitor cells. When RepSox was removed and the Ca^2+^ concentration raised, the cells were able to differentiate normally at any given passage cycle up to one year, suggesting the lack of malignant transformation after RepSox expansion. Furthermore, this finding was not restricted to keratinocytes: with RepSox, newborn and 4-week-old mouse epithelial cells from the salivary gland, tongue, esophagus, bladder, thymus, and cornea all survived expansion for at least 60 days. These cultures maintained their epithelial progenitor cells, evidenced in the production of large holoclone-like clones in 3T3-J2 co-culture and in the expression of epithelial progenitor cell-associated genes (CK14, CK8, CK19 Foxa1, and Sox2) measured by quantitative RT-PCR [65]. With this final addition of RepSox to the cFAD medium and 3T3-J2 feeder techniques that Green pioneered (Figure 2), the milestone of long-term culture of mouse epithelial stem cells was reached.

The two pediatric burn patients saved by cultured epithelial autografts in 1984 represented a clinical and scientific breakthrough, to say nothing of their chance to live full lives. In 2015, another pediatric patient presented with a dire condition known as Junctional Epidermolysis Bullosa (JEB). As a disease class, Epidermolysis Bullosa is characterized by extreme fragility of the skin that leads to frequent blisters despite minimal injury [66]; epidermal autografts grown on collagen sponges had been used to treat facial erosions in decades prior [67].

Tobias Hirsch and collaborators pursued a gene therapy approach for the patient, who had complete epidermal loss on roughly ~60% of the body surface. As usual, the Green protocol began with a biopsy and expansion of primary keratinocytes from the biopsy sample; Hirsch took this a step further and transduced the primary keratinocytes from the biopsy with a retroviral vector. This vector expressed the wildtype cDNA for the *LAMB3* gene, one of three genes jointly responsible for coding the basement membrane protein laminin-332; the mutation of these genes characterizes JEB.

The transgenic keratinocytes maintained consistent percentages of holoclones throughout the mass culture and formed epidermal graft sheets. Ultimately, the treatment team was able to cover the patient’s extant lesions with the transgenic graft sheets. The fully regenerated epidermis spread over time to restore ~80% of the body surface. At biopsies 4, 8, and 21 months after grafting, there was no sign of blistering or erosion and the epidermis had wholly normal morphology [68]. This case represented a novel change to the Green cultured epidermal autograft technique through use of gene therapy, one that not only healed an extant ailment but spurred long-term repair and prevented recurrence.

**Figure 3:**
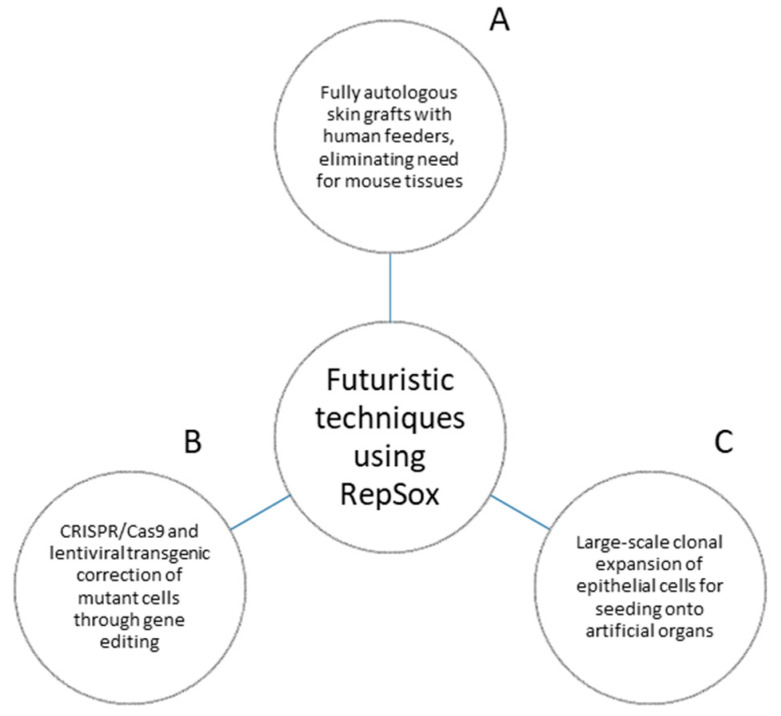
Futuristic techniques using RepSox. (**A**) RepSox allows for fully humanized autologous skin grafts, eliminating reliance upon mouse tissues. (**B**) RepSox enables the growth and maintenance of genetically mutant cells corrected by techniques such as CRISPR/Cas9 genetic editing and lentiviral-mediated transgenes. No current examples of CRISPR/Cas9 used with the Green method exist, and the current most advanced technique is a transgenic approach. Ideally, we can adapt the same system to CRISPR/Cas9 for greater accuracy in the repair of mutant protein expression. (**C**) RepSox enables mass expansion of the vast cell quantities required for large-scale seeding onto artificial organs.

Though the 3T3-J2 cell line has been a mainstay in epithelial stem cell research since its inception, recent developments have shown the feasibility of other approaches. With the use of RepSox, our lab was successful in replacing mouse 3T3-J2 feeder cells with human feeder cells (dermal fibroblasts and preadipocytes) to culture human epidermal keratinocytes. This effectively ended the reliance on 3T3-J2 feeder layers, and allowed for the creation of completely autologous skin grafts with no reliance on mouse tissues [69]. RepSox continues to show potential to enable future scientific approaches (Figure 3).

## 5. Conclusions

Howard Green did not work alone for any of his seminal breakthroughs. He recognized the necessity of collaboration, freely gifting the 3T3-J2 cell line for experimental work. The successors that built upon his legacy were not themselves isolated in their accomplishments, and the whole of epithelial stem cell research is thus a testament to the collective benefits of scientific openness. The progression of gene editing to treat disease, the exploration of biomaterials and engineering for artificial skin, the establishment of long-term epithelial stem/progenitor culture, and the replacement of 3T3-J2 feeders with human feeders for fully autologous skin grafts are all owed to that openness. The field will doubtless look vastly different in another fifty years. Howard Green’s legacy, however, will remain the same.

## Figures and Tables

**Table 1:** Early advancements in epithelial stem cell research by Howard Green and collaborators.

Year	Authors	Innovation
1963	Todaro and Green	Creation of 3T3 cell line from primary culture of Swiss mouse embryonicfibroblasts
1975a	Rheinwald and Green	Growth of keratinizing cell line XB derived from mouse teratoma with 3T3 feeder support
1975b	Rheinwald and Green	Human epidermal keratinocytes serially grown in 3T3 co-culture
1979	Green, Kehinde, Thomas	Single cells cultured into stratified colonies that fuse into graft-viable epithelial sheets, addition of EGF and cholera toxin to culture medium
1984	Gallico III, O’Connor, Compton, Kehinde, Green	Wounds of two pediatric third degree burn patients successfully covered with human cultured epithelial autografts
1989	Rheinwald and Green	Subcloning of 3T3 cells to create 3T3-J2 mouse embryonic fibroblast cell line

**Table 2:** Modern advancements in epithelial stem cell research.

Year	Authors	Innovation
1999	Pellegrini et al.	Preservation of epidermal stem cell holoclones by culturing on fibrin substrate to improve autograft efficiency for full-thickness burn treatment
2000	Horch, Debus, Wagner	“Upside-down” method: cultured human keratinocyte layers grown atop collagen membranes and transplanted cell side down onto nude mice wounds
2009	Ichida et al.	Proof of Sox2 replacement by small molecule RepSox to inhibit TGF-β signaling and drive reprogramming of differentiated cells into iPS cells
2017	Suzuki, Pinto, Senoo	RepSox inhibition of TGF-β signaling enabled long-term p63+ epithelial progenitor expansion
2017	Suzuki, Pinto, Senoo	Replacement of 3T3-J2 mouse feeder cells with human feeder cells in primary human keratinocyte culture for fully autologous skin grafts
2017	Hirsch et al.	Genetic correction of mutant *LAMB3* gene in child with Junctional Epidermolysis Bullosa allowed full epidermal regeneration with transgenic stem cells

## Data Availability

Data sharing is not applicable to this article.
